# Fish do not feel pain and its implications for understanding phenomenal consciousness

**DOI:** 10.1007/s10539-014-9469-4

**Published:** 2014-12-16

**Authors:** Brian Key

**Affiliations:** School of Biomedical Sciences, University of Queensland, Brisbane, 4072 Australia

**Keywords:** Fish, Pain, Phenomenal consciousness, Affective states, Avoidance learning, Neocortex, Pallium

## Abstract

Phenomenal consciousness or the subjective experience of feeling sensory stimuli is fundamental to human existence. Because of the ubiquity of their subjective experiences, humans seem to readily accept the anthropomorphic extension of these mental states to other animals. Humans will typically extrapolate feelings of pain to animals if they respond physiologically and behaviourally to noxious stimuli. The alternative view that fish instead respond to noxious stimuli reflexly and with a limited behavioural repertoire is defended within the context of our current understanding of the neuroanatomy and neurophysiology of mental states. Consequently, a set of fundamental properties of neural tissue necessary for feeling pain or experiencing affective states in vertebrates is proposed. While mammals and birds possess the prerequisite neural architecture for phenomenal consciousness, it is concluded that fish lack these essential characteristics and hence do not feel pain.

## Introduction

There is a belief in some scientific and lay communities that because fish respond behaviourally to noxious stimuli, then *ipso facto*, fish feel pain. Sneddon (2011) clearly articulates the logic by stating: “to explore the possibility of pain perception in nonhumans we use indirect measures similar to those used for human infants who cannot convey whether they are in pain. We measure physiological responses (e.g., cardiovascular) and behavioral changes (e.g. withdrawal) to assess whether a tissue-damaging event is painful to an animal”. In some cases, the inference that fish have affective states arises because of conflation of nociception with pain (Demski 2013; Kittilsen 2013; Malafoglia et al. 2013). Interestingly, sometimes the difference between nociception and pain is recognized but it is still considered safer to err on the side of caution and accept that fish feel pain (Jones 2013). Unfortunately, endowing fish with the subjective ability to experience pain is typically undertaken without reference to its neurophysiological bases (Rose 2002, 2007; Browman and Skiftesvik 2011).

Before interrogating the issue of fish feeling pain and its implications for phenomenal consciousness, I will briefly define several key terms. When I refer to fish it is with the knowledge that this is a highly diverse paraphyletic group consisting of ~30,000 species. Since most of the behavioural and neuroanatomical investigations discussed here have been undertaken only on a small number of ray-finned fish, there is considerable extrapolation involved when I use the generic term fish. A noxious stimulus is one that is considered to be physically harmful to an animal without reference to feelings. For example, excessive heat, a skin incision, toxic chemical exposure and extreme mechanical pressure are all stimuli that can perturb normal tissue morphology, and are hence considered to be noxious. Nociception is referred to as the neurobiological processes associated with the activation of peripheral sensory neurons and their upstream neural pathways by noxious stimuli in the absence of conscious feeling. In contrast, pain is the subjective experience of feeling a noxious stimulus (however, in certain central neuropathies in humans it can arise without external stimuli). The subjective “feeling” associated with a sensory stimulus is also referred to as a “quale” or “phenomenal consciousness” (Kanai and Tsuchiya 2012). Given the above, I acknowledge the tautology in the manuscript’s title since the word “pain” is already defined as “to feel a noxious stimuli”. However, the phrase “feel pain” within the title was chosen to over-emphasize the subjective or qualitative nature of pain.

One of the main proponents in the literature of the thesis that fish do not feel pain has been John D. Rose. In a series of comprehensive articles (Rose 2002, 2007; Rose et al. 2014) it was argued that fish do not experience the sensation of pain. Anthropomorphism was considered as a hindrance to understanding the underlying causes of behavioural responses of animals to sensory stimuli (Rose 2002, 2007). Rose advocated attention to the evolution, development and organization of the nervous system in order to understand fish behaviour. He initially drew attention to three key issues (Rose 2002). First, behavioural responses to sensory stimuli must be distinguished from psychological experiences. Second, the cerebral cortex in humans is fundamental for the awareness of sensory stimuli. Third, fish lack a cerebral cortex or its homologue and hence cannot experience pain or fear. In 2007, Rose highlighted the problems of anthropomorphic thinking in respect to fish behaviour and how it influenced welfare issues. He stressed that pain and emotion were not primitive feelings that arose early in vertebrate evolution but were rather more recent acquisitions, associated with the emergence of the cerebral cortex (Rose 2007). In 2014, Rose et al. (2014) rebutted experimental evidence supposedly supporting claims that fish feel pain. They demonstrated deficiencies in methodological approaches and highlighted problems in concluding pain experience from behavioural responses. Moreover, they recognized that teleosts typically lack nociceptors responsible for transmission of pain but instead have an abundance of A-delta fibres that are most likely subserving escape and avoidance responses rather than the experience of pain.

Despite the work of Rose et al. (Rose 2002, 2007; Rose et al. 2014) there remains a strong trend in the literature to bestow fish with the ability to feel pain and to experience fear and other emotions. The alternate view that fish do not feel pain or experience affective states needs more careful consideration, particularly as it has consequences for understanding the neuroanatomical basis of phenomenal consciousness. Here I consolidate the arguments for why fish are believed to feel pain into six main reasons. By undertaking a deeper analysis of the behavioural observations in the light of our understanding of neurophysiology and neuroanatomy, I subsequently propose that it is more plausible and probable to reason that fish do not feel pain. Concluding that fish do not feel pain affords an opportunity to define the basic architectural properties of the neural circuitry necessary for phenomenal consciousness through comparisons of fish and mammalian neuroanatomies. These properties then provide a simple tool for assessing the likelihood that a vertebrate animal will experience “feelings” such as pain.

## What are the reasons for the anthropomorphic view that fish feel pain?

There are six principal reasons that account for why some people believe that fish feel pain. One, fish demonstrate behaviours consistent with the way humans might react to noxious stimuli that cause pain. For example, fish will either attempt to rapidly escape or display anomalous behaviour (Reilly et al. 2008) in response to noxious stimuli, such as electric shock or a chemical irritant. Two, medicating fish with an analgesic (a drug that attenuates pain in humans) reduces the escape response to electric shock (Sneddon 2003; Sneddon et al. 2003; Jones et al. 2012). Three, fish display classic physiological indicators of stress such as increased ventilation and heart rate and elevated blood levels of the stress hormone cortisol during and after exposure to supposedly stressful stimuli (Reilly et al. 2008; Filk et al. 2006; Wolkers et al. 2013). Four, fish have nociceptive nerve fibres and have increased neural activity in the spinal cord, hindbrain and pallium that is specifically associated with a noxious stimulus (Dunlop and Laming 2005). Five, fish can be trained to associate a neutral signal with an impending noxious stimulus and so learn to escape prior to experiencing the noxious stimuli (Dunlop et al. 2006). Six, it is evolutionarily advantageous to feel pain in order to prevent body injury.

### Behavioural responses to noxious stimuli are not necessarily evidence of pain

It is common to attribute inner mental states or feelings to organisms or even inanimate objects on the basis of observed behavior. When a noxious stimulus is applied either to the plantar surface of the human foot, or directly to the nerves innervating this region, there is a reflex withdrawal of the lower limb involving contraction of the hip and knee flexors, and relaxation of the extensors. This reflex is protective and enables the rapid removal of the limb from a harmful stimulus. Complete spinal cord injury patients, who lack sensations arising from the lower limb, continue to exhibit the withdrawal flexion reflex (Dimitrijevic and Nathan 1968). Thus, reflexes are neither good evidence for, nor a measure of feeling pain. Nonetheless, simple reflex behaviours in response to noxious stimuli continue to be inappropriately used to suggest that fish feel pain.

Fish exhibit behavioural responses to somatosensory stimulation from a very early stage of development. For example, within the first few days of fertilisation, zebrafish embryos response to touch by initially exhibiting a twitch of their tail, and then slightly later in development, by a few strokes of their tail that elicits a short burst of swimming. While it is tempting to attribute feelings to these embryos, it must be remembered that the telencephalon is not yet morphologically distinct when the touch response first appears at around 21 h post-fertilisation (Hjorth and Key 2001; Saint-Amant 2006). Moreover, a lesion to the anterior spinal cord, that isolates the cord from the brain, does not affect the execution of the touch-induced swimming escape response (Pietri et al. 2009). Thus, simple reflex escape behaviours of fish that can be activated by somatosensory stimuli are best not used as evidence for fish experiencing phenomenal consciousness.

It is important here to draw attention to the fact that pain in humans arises in the forebrain, and is distinct from unconscious behavioural responses mediated by lower brain levels. The forebrain also plays an essential role in pain perception in other mammals. This is elegantly illustrated in a rat model of pain that uses injection of a dilute solution of formalin into the paw. This chemical irritant induces a variety of body movements such as paw shaking, licking and grooming. Animals also exhibit a protective response and attempt to reduce contact of the affected limb with the floor. These behaviours are sometimes considered as indicators of pain. However, rats continued to exhibit such behavioural responses following surgical decerebration (Matthies and Franklin 1992, 1995). One interpretation of these results is that pain is actually experienced in the brainstem, and not in the forebrain in rats. However, this is most unlikely given that systemic administration of an analgesic (morphine) does not attenuate behavioural responses to formalin in decerebrated animals. Morphine was only effective in inhibiting behaviours when connections between the forebrain and brainstem were left intact in sham-operated rats (Matthies and Franklin 1992). Moreover, local application of morphine into either the somatosensory, prefrontal orbital or agranular insular cortices attenuates behavioural responses in the formalin pain model in rats (Soto-Moyano et al. 1988; Xie et al. 2004). Thus, morphine is active in the rat forebrain, which is consistent with it modulating the subjective experience of the noxious stimuli, as in humans (Jones et al. 1991; Taylor et al. 2013).

Fish are known to swim away from noxious electric shock and this behavioural response has been used to indicate that these animals feel pain. However, this interpretation is simplistic and can be dismissed given the extensive evidence that fish continue to exhibit escape behaviour following ablation of the entire telencephalon (Hainsworth et al. 1967; Davis et al. 1976). Forebrainless fish display no clear evidence of deficits in normal behaviours. For example, forebrainless fish continue to flee from capture by a small fish net with similar locomotor agility as their unoperated counterparts (Kaplan and Aronson 1967). The ability to escape or respond to an electric shock is unaffected by removal of either the forebrain or telencephalon in goldfish (Hainsworth et al. 1967; Savage 1969; Portavella et al. 2004a, b) or telencephalon in *Tilapia mossambica* (Overmier and Gross 1974).

In summary, the idea that fish flee noxious stimuli because they experience phenomenal consciousness (feel pain) is not the best explanation for this behaviour. It is more probable that fish demonstrate these behaviours because they have evolved innate reflexes associated with specific spinal and sub-telencephalic neural circuits.

### Modification of behaviour with drugs does not necessarily demonstrate pain

It has been proposed that if an animal’s behavioural response to a noxious stimulus is attenuated following administration of a drug known to be an analgesic in humans, then it  is likely that the animal can feel pain. However, it needs to be pointed out that analgesics can be active at multiple sites in the neuroanatomical pathways associated with noxious stimuli. If an analgesic blocks or reduces neural activity in the spinal cord (Yaksh and Rudy 1976) it can subsequently attenuate neural responses in the brainstem and telencephalon. Similarly, if an analgesic works at the level of the brainstem it can modulate both brainstem and higher-order brain responses (Pert and Yaksh 1975). If an analgesic is active at the level of the telencephalon and reduces behavioural responses (Xie et al. 2004) then the animal, at least, has the possibility of feeling a noxious stimulus as painful (however this interpretation is dependent first, on the behaviour being non-reflexive and second, on the existence of the necessary neural hardware; see below). At present, the inference that fish feel pain because behavioural responses to noxious stimuli are attenuated following systemic administration of morphine (Sneddon 2003) is weak, particularly given that both the site of action as well as the physiological role of this drug in fish are unknown.

### Physiological stress is not pain

Physiological stress as determined by plasma cortisol levels and opercula beat rate have been used as indicators of feeling pain by fish (Chandroo et al. 2004; Braithwaite and Boulcott 2007; Scott Weber 2011). The underlying assumption in these cases is that if a fish is exposed to a stimulus that triggers both increased cortisol and behavioural responses, then that fish must be consciously feeling that stimulus as a mental state such as fear and/or pain. If pain was felt by a fish exposed to a physiological stressor, and cortisol was an indicator of the level of discomfort that fish experienced, then one would predict increased cortisol levels in fish as a noxious stimulus was increased. However, this does not appear to be the case. There is no relationship between the apparent “stressful” stimulus and the level of cortisol in fish (Roques et al. 2010). Even when the stimulus causes increased behavioural responses there was no relationship to the level of plasma cortisol. The cortisol response to increased stress seems to be highly variable (Fatira et al. 2014; Quillet et al. 2014) and context specific (Manek et al. 2014). Surprisingly, exposure to multiple stressors simultaneously can lead to decreased rather than an expected increase in cortisol levels (Manek et al. 2014). Thus, changes in cortisol levels in fish are better explained by autonomic responses to external environmental stresses rather than by internally generated mental states such as fear or pain.

### Brain activity in response to noxious activity is not equivalent to pain

It has been proposed that fish can feel pain both because they have peripheral nociceptors and because neural responses to noxious stimuli have been recorded in the spinal cord, cerebellum, tectum and telencephalon of fish (Sneddon 2004; Dunlop and Laming 2005). Nordgreen et al. (2007) reported neural activity in the telencephalon following electrical stimulation of the tail of Atlanic salmon. While these authors indicated that this activity is a necessary prerequisite for feeling pain, they realised that it does not necessarily provide evidence for the ability of fish to feel pain. Unfortunately, the neuroanatomical localisation of electrical activity recorded in the telencephalon has not been described. If activity was recorded in the dorsal pallium (homologous to the neocortex; Mueller and Wullimann 2009; Mueller et al. 2011) of the telencephalon, it would, at least, provide some phylogenetic insight into neural pathways underlying nociception. It would not, however, be evidence of pain or emotion.

### Associative learning using noxious stimuli is possible without feeling pain

Considering the problems with using simple behavioural responses to noxious stimuli as a measure of pain sensation, avoidance learning has instead been adopted as a means for assessing pain in animals. Rats can easily learn to avoid locations in a cage where electric shocks are delivered and to push a lever that terminates the shock. This learning is viewed as requiring the animal to initially decipher the stimulus (i.e. feeling the stimulus as painful and assessing the intensity using the cerebral cortex; Baastrup et al. 2010) and then to plan and perform a relatively complex motor task (Vierck 2006). Higher-level brain activity (involving the cerebrum) is essential for avoidance learning since decerebrate rats fail to learn to avoid electric shock (Vierck 2006). Interestingly, rats exhibit an escape response substantially faster than a brainstem reflex (such as paw licking or jumping) in response to a noxious stimulus (Vierck 2006). In addition, the rat threshold for escape response from cold temperatures is approximately 16 °C whereas the threshold for brainstem reflexes is <5 °C (Vierck 2006). These comparisons between brainstem reflexes and higher-level escape responses suggest that the cerebrum quickly perceives noxious stimuli as potentially harmful before they are actually physically damaging. Taken together, these results are consistent with rats feeling pain.

Operant conditioning with negative reinforcement demonstrates that fish can also learn to associate a conditioned stimulus (light cue) with an impending unconditioned stimulus (electric shock) administered in one chamber of a two-chamber holding tank (Hurtado-Parrado 2010). Fish typically learn to terminate their exposure to the electric shock by escaping to the chamber where the shock is not present. With more and more trials, the fish learn to associate the light stimulus with the temporally delayed electric shock and hence begin to escape prior to the delivery of the shock. However, as pointed out above, the escape response in fish is a reflex behaviour and does not equate to the more complex escape routines used in rodent models of pain (Cain et al. 2010). Thus, the better explanation is that fish reflexively associate the stimulus with the shock.

It has been reasoned that if a behavioural response was modifiable under different circumstances, then it was not a reflex. This vague distinction between reflex and non-reflexive (or flexible) behaviours in fish relies on the notion that higher-level brain activity was associated with the latter and not the former (Dunlop et al. 2006; Braithwaite et al. 2013). Evidence for this activity was purported to come from numerous observations that telencephalon ablation perturbed avoidance learning in fish. However, it has been consistently reported that although avoidance learning by fish is perturbed by full or partial forebrain ablations, these animals continue to exhibit escape responses (and many continue to learn to avoid) as a result of electric shock (Hainsworth et al. 1967; Kaplan and Aronson 1967; Savage 1968; Overmier and Gross 1974; Flood et al. 1976; Overmier and Papini 1985; Portavella et al. 2003, 2004a, b; Portavella and Vargas 2005; Vargas et al. 2009). Thus, forebrainless fish are still able to either escape from, or learn (albeit more slowly) to avoid, an electric shock. Fish with, or without, the forebrain had similar latencies of escape. Escape latency was the time taken for a fish to escape from the chamber once it received a shock. Clearly, the forebrain was not needed for fish to exhibit escape behaviour, but it was important for learning the association between the light and the unconditioned stimulus (shock).

Taken together, the above results demonstrate that the escape responses used in the avoidance learning paradigms for fish involve sub-forebrain regions associated with instinctive and/or reflexive behaviours. Thus, the avoidance learning paradigms typically used in fish studies are more informative about learning processes in fish, then about the sensation of pain experienced by these animals. It is most likely that the sorts of avoidance learning exhibited to date in fish studies is better explained by innate neural circuitry mediating reflex behaviour.

### Pain is not essential for reducing injury

The idea that nociception has an evolutionary survival advantage for animals is well established in the scientific literature (Kavaliers 1988). However, the significance of feeling pain in animals is less well understood since the nociception-pain axis has not been carefully interrogated. It has been assumed that pain enables animals to adopt longer-term protective behaviours in order to facilitate tissue repair and to prevent compounding injuries (Bolles and Faneslow 1980).

If fish were to feel pain then one would, at least, expect them to exhibit a longer-term protective response to injury. The fins of fish are densely innervated by sensory axons and are one of the most highly sensitive regions of the fish body surface to noxious stimulation (Chervova 1997). If fish were experiencing pain, and if pain was serving a protective function, then fish should respond to fin injury either by not using that fin or by altering swimming behaviour until the injury was repaired. However, after either partial or complete tail fin amputation, fish show no evidence of protecting their fins by reducing their swimming behaviour; they are instead quite capable of swimming continuously against a current (Fu et al. 2013). These observations are also consistent with the normal behaviour of fish with bacterial tail or fin rot. This disease causes progressive erosion of the affected fins/tail and yet these fish swim and eat normally. The consensus in the fish welfare literature is that fin rot, despite its ability to cause loss of most of the tail fin, does not affect the behaviour of fish. These animals continue to eat and swim like their healthy counterparts (Ellis et al. 2008). The most plausible interpretation of these observations is that fish do not modulate long-term behaviour in order to allow injury repair. This conclusion is more consistent with fish not feeling pain.

## What is the neural basis of pain?

I have suggested above that the behavioural responses of fish to noxious stimuli is best explained by sub-telencephalic reflexes mediated by innate neural circuits rather then by fish experiencing phenomenal consciousness. By accepting this argument it now becomes possible to better address the necessary anatomical prerequisites underlying phenomenal consciousness. All chordates possess a central nervous system consisting of an enlarged anterior end and a posterior cord-like structure. The differences in neuroanatomy that have emerged during evolution within this phylum reflect specialised functions (Butler 2000). While the posterior cord has typically preserved a simple morphology that subserves basic locomotor behaviours, the rostral nervous system has instead undergone extensive structural modifications that have led to devise functional consequences. For instance, the evolution of the neocortex in humans has allowed us to experience our environment through subjective mental states such as pain, smell, hearing and vision. By understanding how our environment subjectively “feels” it has become possible for humans to appreciate and predict how other people would respond in certain situations. Consequently by manipulating our environment we are able to affect the behaviour of others to achieve specific outcomes. The human neocortex is particularly adept at this function and it is clearly an important driving force in our cultural evolution.

What is so unique about the cortex that enables inner mental states? First, the cortex is parcellated into discrete anatomically structures or cortical areas that process information related to specific functions. It is estimated that there are about 200 cortical areas in humans (Kaas 2012). For instance, the cortical visual system consists of over a dozen distinct regions with diverse subfunctions that are strongly interconnected by reciprocal axon pathways. One of the defining features of these subregions is that they become simultaneously active. Both recurrent activity and binding of neural activity across cortical regions are believed to be essential prerequisites for the subjective experience of vision (Sillito et al. 2006; Pollen 2011; Koivisto and Silvanto 2012). It has been shown that when neural processing of recurrent signalling from higher cortical regions entering the V1 visual cortex is perturbed by transcranial magnetic stimulation, the subjective awareness of a visual stimulus is disrupted (Koivisto et al. 2010, 2011; Jacobs et al. 2012; Railo and Koivisto 2012; Avanzini et al. 2013).

The subregionalisation of the neocortex also allows the formation of spatial maps of the sensory world, such as those associated with the representations of the surface of the body or the visual field. These topographical maps are important for the multiscale processing of sensory information (Kaas 1997; Thivierge and Marcus 2007). Variation in the size of the maps alters the sensitivity of responses to stimuli while spatial segregation of neurons responding to selective parts of a stimulus allows for finer perceptual discrimination. Painful and non-painful somatosensory stimuli are topographically mapped to overlying regions in the primary somatosensory cortex (SI) in humans (Mancini et al. 2012). These results are consistent with the known point-to-point topography from the body surface to SI (called somatotopy) that underlies spatial acuity. However, by using high resolution mapping in the squirrel monkey SI (sub-millimetre level) it was revealed that there were slight differences in the localisation of different somatosensory modalities (Chen et al. 2001). This slight physical separation of cortical neurons responding to different peripheral stimuli suggests that differences in the subjective quality of somatosensory sensations may arise as early as in SI. Somatotopic maps for painful stimuli are also present in the human SII and insular cortices (Baumgartner et al. 2010). Interestingly, different qualities of painful stimuli (such as heat and pinprick) are more distinctly mapped topographically to different regions of SII and the insular cortex than in SI. Similarly, painful and non-painful stimuli are mapped to separate regions in human SII (Torquati et al. 2005). This separation of cortical processing of heat and tactile stimuli within different cortical areas has also been observed in non-human primates (Chen et al. 2011). These multiple neural maps suggests that SII and the insular cortex play important roles in discriminating differences in the subjective quality of somatosensory stimuli, particularly painful from non-painful (Tommerdahl et al. 1996; Baumgartner et al. 2010; Chen et al. 2011; Mazzola et al. 2012). This idea is supported by evidence from direct electrical stimulation of discrete areas in the human insular cortex (Afif et al. 2010).

Second, the cortex is a laminated structure that enables the efficient processing and integration of different types of neural information by unique subpopulations of neurons (Schubert et al. 2007; Maier et al. 2010; Larkum 2013). Lamination appears to facilitate complex wiring patterns during development. If two populations of neurons were randomly distributed within a specific brain region and incoming axons were required to synapse with only one subpopulation, then those axons would need to rely on stochastic and hence error-prone searching to complete wiring. On the other hand, when similar neurons are partitioned together in a single lamina then a small set of molecular cues is able to guide axons with high precision to their appropriate post-synaptic target. Two principal afferent inputs (from the neocortex itself, and the thalamus) enter the neocortex and separately innervate distinct layers (Nieuwenhuys 1994). The main thalamic fibres terminate densely in layer IV (called the granular layer) while the neocortical fibres innervate different pyramidal neurons in layers I–III (supragranular layers) (Opris 2013). By selectively ablating Pax6, a developmentally significant patterning gene, in the cortex of mice it is possible to disrupt the laminar organisation of this structure (Tuoc et al. 2009). This altered cortical layering causes neurological deficits that are similar to those observed in humans with Pax6 haploinsufficiency (Tuoc et al. 2009) and provides strong experimental evidence of the importance of lamination to cortical function. A number of human brain disorders involve defects in cortical lamination that are detrimental to brain function (Guerrini et al. 2008; Guerrini and Parrini 2010; Bozzi et al. 2012).

Third, lamination facilitates the economical establishment of microcircuitry between neurons processing different properties of the stimulus. A vertical canonical microcircuit is established which leads to the emergence of functionally interconnected columns and minicolumns of neurons (Mountcastle 1997). For example, a hexagonal column in the primate somatosensory cortex is about 400 μm in width and contains populations of neurons that respond to the same stimulus (e.g. light touch or joint stimulation) arising from a specific topographical zone of the body. Columns can be associated with processing information related to a specific function (e.g. “visual tracking” and “arm reach” columns in the parietal cortex; Kass 2012). Each column itself consists of minicolumns (80–100 neurons) that are ~30–50 μm in diameter and interconnected by short-range horizontal processes (Buxhoeveden and Casanova 2002). While columns are most clearly distinguished in the sensory and motor cortices of primates, minicolumns appear to be ubiquitous in all animals with a neocortex (Kaas 2012). Minicolumns have a small receptive field within the larger receptive field of the column. The correlated activity in the fine-scale networks of minicolumns produces concentrated bursts of neural activity that may enable the cortex to transmit signals in the face of background noise (Ohiorhenuan et al. 2010). The function of the cortex seems to depend on the ability of canonical circuitry within the minicolumns to rapidly switch from feedforward to feedback processing between layers. During learned tasks in responses to cues in the awake monkey, information flows from layer 4 to layer 2/3 and then down to layer 5 in a feedforward loop in the temporal neocortex (Takeuchi et al. 2011; Bastos et al. 2012). This is followed shortly afterwards by a feedback loop from layer 5 to layer 2/3. Correlated firing of layer 2/3 and layer 5 neurons in minicolumns occurs during decision making in the monkey prefrontal cortex, an area responsible for executive control in primates (Opris et al. 2012). The accuracy of error-prone tasks was increased when layer 5 neurons were artificially stimulated by activity recorded during successful task execution. These results provide evidence for the role of the minicolumn as the fundamental processing unit of the neocortex associated with higher order behaviour (Bastos et al. 2012; Opris et al. 2012).

In summary, the unique morphology of the mammalian cortex facilitates multiscale processing of sensory information. Initially there is course scaling at the level of gross anatomical cortical regions specialising, for example, in processing of visual or somatosensory information. Some of these regions are then topographically mapped in order to preserve spatial relationships and facilitate selective processing of specific sensory features. Importantly, to preserve the holistic quality of a sensory stimulus, these subregions are strongly interconnected via axon pathways that create synchronized re-entrant loops of neural activity. Cortical regions are laminated which supports finer scale sensitivity in the processing of specific features. Finally, canonical microcircuits (minicolumns) bridge across layers to enhance signal contrast (Casanova 2010). Local connectivity between minicolumns enables the lowest level of stimulus binding that contributes to the holistic nature of the stimulus (Buxhoeveden and Casanova 2002).

I propose that only animals possessing the above neuroanatomical features (i.e. discrete cortical sensory regions, topographical maps, multiple cortical layers, columns/minicolumns and strong local and long-range interconnections), or their functionally analogous counterparts, have the necessary morphological prerequisites for experiencing subjective inner mental states such as pain. It has been argued that since the avian pallium is non-laminated, and yet these animals exhibit high levels of cognitive ability and behaviours rivalling those of primates, that lamination is not an essential prerequisite for consciousness (Gunturkun 2005; Kirsch et al. 2008; Gunturkun 2012; Veit and Nieder 2013). However, the classic view of the organisation of the avian telencephalon has been revised and previous subpallial regions are now recognised as pallial in nature (Shimizu 2009). Careful examination of pallial neuroanatomy has further revealed that distinct regions of the avian pallium act like layers of the neocortex (Dugas-Ford et al. 2012). Moreover, columnar processing units appear to operate across these brain regions in the processing of sensory and motor information (Jarvis et al. 2013). When this is combined with complex parcellation, the presence of topographical maps and strong interconnectivity in the avian pallium (Shimizu et al. 1995; Shimizu and Bowers 1999; Bingman and Able 2002; Manger et al. 2002; Nguyen et al. 2004; Watanabe and Masuda 2010), it appears that birds possess the necessary neural machinery for phenomenal consciousness.

The pallium of fish is non-laminated. It is partitioned into five broad nuclear regions (dorsomedial, dorsolateral, dorsodorsal, dorsoposterior and ventral; Northcutt 2011). While the dorsodorsal pallium is believed to be homologous to the neocortex there remains some controversy as to the definitive homology between these structures (Echleter and Saidel 1981; Northcutt 2008; Braford 2009; Northcutt 2011). There is converging evidence from electrophysiological recordings (Precht et al. 1998; Saidel et al. 2001; Northcutt et al. 2004) and neuroanatomical tracing (Yamamoto and Ito 2008) that, unlike in the neocortex, sensory information such as visual input, is diffusely processed across the fish dorsal pallium, and certainly not localised to multiple interconnected areas that are topographically mapped (Giassi et al. 2012). Evidence is also lacking for canonical microcircuitry subserving fine scale processing of sensory information in the dorsal pallium. This lack of contrast in signal processing does not support the ability of the fish pallium to differentiate sensory modalities with sufficient resolution to allow the emergence of distinct feelings for different sensory modalities.

It has been suggested that sub-forebrain structures in fish may somehow take over the function of phenomenal consciousness in the neocortex. While parcellated sensory processing, laminated cytoarchitecture and columnar-like modules are present in the mid- and hindbrains of some fish (Meek 1983; Krahe and Maler 2014), these structures lack the necessary local and long-range feedforward and recurrent pathways associated with information binding underlying phenomenal consciousness (Baars et al. 2013). Instead, the vertebrate midbrain optic tectum has conserved structural features across a variety of species such as fish, frogs, birds and mammals that subserve common functionalities (e.g. orienting, direction-sensitivity, and spatial relationships; Ingle 1973). Furthermore, while ablation of the tecta perturbs visual function, startle responses in tectumless fish are preserved (Yager et al. 1977; Roeser and Naier 2003). Thus, the tectum is not needed to respond to somatosensory stimuli and certainly does not possess novel circuitry responsible for pain. On the basis of our current understanding of the structure and function of the “fish” brain, it most likely that fish do not have the necessary neural machinery for phenomenal consciousness.

In summary, I have demonstrated how misleading it is to infer that fish have feelings on the basis of behavioural responses to sensory stimulation. It is essential that our anthropomorphic tendencies to bestow animals with feelings does not hinder the progress of scientific enquiry into the evolution of phenomenal consciousness. I propose that there are a number of fundamental neural building blocks that are necessary prerequisites for phenomenal consciousness in the vertebrate lineage. The possession of this hardware sets the minimal requirements for the sensation of noxious stimuli as painful. The idea that other neural architectures that have been specifically wired for fundamentally different functions in vertebrates (such as the mid- and hindbrains) could also subserve pain in fish is incongruent with evolutionary biology and neuroscience. While there is some degree of plasticity of function in the mammalian neocortex (Kupers and Pitto 2013), the very notion that either the fish tectum as well as the mid- and hindbrain reticular formations (that are reciprocally interconnected with the tectum; Perez-Perez et al. 2003; Luque et al. 2005) has some hidden neural circuitry that allows for the processing of somatosensory inputs into discrete feelings of pinprick, heat, cold, scratch, cutting and stabbing is difficult to defend.
